# Severe liver fibrosis in the HCV cure era: Major effects of social vulnerability, diabetes, and unhealthy behaviors

**DOI:** 10.1016/j.jhepr.2022.100481

**Published:** 2022-03-30

**Authors:** Patrizia Carrieri, Fabrice Carrat, Vincent Di Beo, Marc Bourlière, Tangui Barré, Victor De Ledinghen, Georges-Philippe Pageaux, Morgane Bureau, Carole Cagnot, Céline Dorival, Elisabeth Delarocque-Astagneau, Fabienne Marcellin, Stanislas Pol, Hélène Fontaine, Camelia Protopopescu, ANRS CO22 HEPATHER study group

**Affiliations:** 1 Aix Marseille Univ, Inserm, IRD, SESSTIM, Sciences Economiques & Sociales de la Santé & Traitement de l’Information Médicale, ISSPAM, Marseille, France; 2 INSERM, Institut Pierre Louis d’Epidémiologie et de Santé Publique, Sorbonne Université, Paris, France; 3 Unité de Santé Publique, Assistance Publique - Hôpitaux de Paris, Hôpital Saint-Antoine, Paris, France; 4 Hôpital St Joseph, Service d'Hépato-Gastroentérologie, Marseille, France; 5 Hepatology Unit, University Hospital Bordeaux and INSERM U-1053, Bordeaux University, Pessac, France; 6 CHU Saint-Eloi, Département d’hépato-gastroentérologie et de transplantation hépatique, Université de Montpellier, Montpellier, France; 7 ANRS MIE (France Recherche Nord & Sud Sida-HIV Hépatites, Maladies Infectieuses Emergentes), Paris, France; 8 Université Paris-Saclay, UVSQ, Inserm, CESP, Team Anti-infective Evasion and Pharmacoepidemiology, 78180 Montigny, France; 9 AP-HP, GHU Paris-Saclay University, Raymond Poincaré Hospital, Epidemiology and Public Health Department, 92380 Garches, France; 10 Assistance Publique - Hôpitaux de Paris, Hôpital Cochin, Unité d'Hépatologie, Paris, France; 11 Université Paris Descartes, INSERM U-1223, Institut Pasteur, Paris, France

**Keywords:** coffee, direct-acting antivirals, FIB-4, hepatitis C, alcohol, social status, diabetes, socio-behavioral factors, ALT, alanine aminotransferase, aOR, adjusted odds ratio, DAA, direct-acting antiviral, FIB-4, fibrosis-4, HCC, hepatocellular carcinoma, OR, odds ratio, PAF, population attributable fraction

## Abstract

**Background & Aims:**

After HCV cure, not all patients achieve significant liver fibrosis regression. We explored the effects of clinical and socio-behavioral factors on liver fibrosis, before and after HCV cure with direct-acting antivirals.

**Methods:**

We analyzed data from the ongoing ANRS CO22 HEPATHER cohort, which prospectively collects clinical and socio-behavioral data on HCV-infected patients. Mixed-effects logistic regression models helped identify predictors of longitudinal measures of severe liver fibrosis, defined as a fibrosis-4 index >3.25. We also estimated the adjusted population attributable fractions (PAFs) for modifiable risk factors.

**Results:**

Among the 9,692 study patients (accounting for 24,687 visits over 4 years of follow-up, 48.5% of which were post-HCV cure), 26% had severe fibrosis at enrolment. After multivariable adjustment, HCV-cured patients had an 87% lower risk of severe fibrosis. An inverse dose-response relationship was found for coffee consumption, with the risk of severe fibrosis diminishing by 58% per additional cup/day (adjusted odds ratio (aOR 0.42; 95% CI 0.38-0.46). Unemployment, low educational level, and diabetes were associated with a higher severe fibrosis risk (aOR 1.69; 95% CI 1.32-2.16, aOR 1.50; 95% CI 1.20-1.86, and aOR 4.27; 95% CI 3.15-5.77, respectively). Severe fibrosis risk was 3.6/4.6-fold higher in individuals with previous/current unhealthy alcohol use than in abstinent patients. All these associations remained valid after HCV cure. The risk factors accounting for the greatest severe fibrosis burden were unemployment, low education level, and diabetes (PAFs: 29%, 21%, and 17%, respectively).

**Conclusions:**

Monitoring liver fibrosis after HCV cure is crucial for patients with low socioeconomic status, previous/current unhealthy alcohol use, and diabetes. Innovative HCV care models for the most socially vulnerable individuals and interventions for healthier lifestyles are needed to reinforce the positive effects of HCV cure on liver health.

**Lay summary:**

After hepatitis C virus (HCV) cure, not all patients achieve significant liver fibrosis regression. Herein, we studied the effects of clinical and socio-behavioral factors on the risk of severe liver fibrosis. Coffee consumption was strongly inversely associated with severe fibrosis, while diabetes, previous and current unhealthy alcohol use were associated with a 4.3-, 3.6- and 4.6-fold higher risk of severe fibrosis, respectively. Unemployment and low educational level were also associated with a higher risk of severe fibrosis. All these associations remained valid after HCV cure. These results demonstrate the need to continue liver fibrosis monitoring in at-risk groups, and to facilitate healthier lifestyles after HCV cure as a clinical and public health priority.

## Highlights

• Liver fibrosis assessment is a key prognostic tool in the hepatitis C cure era.
• Significant liver fibrosis regression does not always occur after hepatitis C cure.
• Coffee intake displays protective effects on severe fibrosis even after HCV cure.
• Social vulnerability, diabetes, and unhealthy alcohol use predict severe fibrosis.
• Socio-behavioral factors are associated with severe fibrosis even after HCV cure.

## Graphical abstract

**Figure:**
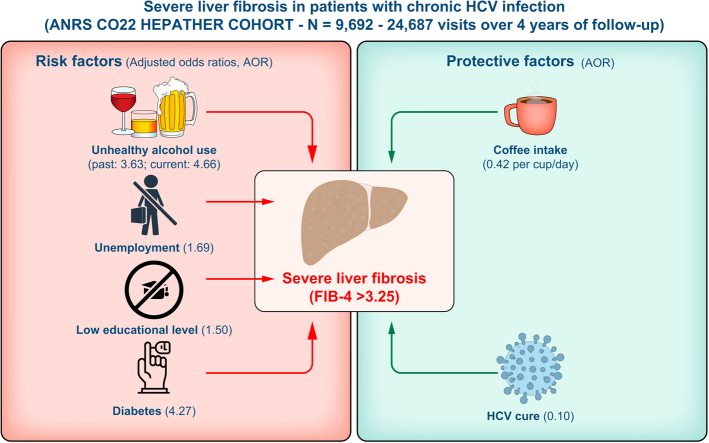

## Introduction

Of the estimated 71 million people infected with HCV worldwide,^1^ 75-85% have chronic infection. This constitutes a silent underdiagnosed epidemic, especially as its early stages are asymptomatic.^1^ If not cured, chronic infection can lead to liver fibrosis progression, cirrhosis, liver cancer, and other liver disease-associated complications.^2^

HCV cure with direct-acting antiviral (DAA) treatments has contributed to a dramatic reduction in the risk of end-stage liver disease-associated complications in recent years.^3^ However, clinical progression of liver disease after HCV cure can vary according to the severity of the disease.^4^ This is why evaluating fibrosis and/or cirrhosis is a key prognostic tool in the HCV cure era.

Although regression of both inflammation and liver fibrosis is expected after HCV cure,^5^ not all patients obtain these benefits,^6^ suggesting that other factors may influence the stage and the evolution of liver fibrosis after HCV cure.

Late HCV diagnosis and advanced liver disease at treatment initiation have been associated with liver fibrosis progression.^7^ Other specific behaviors and lifestyles may also have a considerable effect on liver fibrosis before and after HCV cure. Among these, coffee consumption and alcohol abstinence or very low alcohol consumption are known to have anti-inflammatory effects and/or anti-fibrotic properties.^8^ Although the effects of unhealthy alcohol use on liver fibrosis are widely documented in people living with HCV,^9^ results showing its effect on liver fibrosis after HCV cure in the DAA era are lacking. With regard to coffee, elevated consumption was recently associated with slower liver disease progression (including reduced hepatocellular carcinoma (HCC) incidence in patients with hepatitis C, alcohol-related liver disease, or non-alcoholic fatty liver disease^10^^,^^11^) and with reduced mortality (in HIV-HCV co-infected patients).^12^

Social vulnerability plays a major role in shaping people’s health; this is also observed in the field of liver disease in terms of prevalence, outcomes and access to care. However, data about the effect of social vulnerability on liver-related outcomes before and after HCV cure remain sparse.^13^

The French nationwide ANRS CO22 HEPATHER cohort study of chronic HCV-infected patients gave us the opportunity to conduct a longitudinal exploration of the effects of antifibrotic lifestyle-related factors (in particular coffee consumption), specific addictive behaviors (tobacco smoking, cannabis use and unhealthy alcohol use), and social conditions (employment, educational level) on severe liver fibrosis in chronic HCV-infected patients. We also aimed to verify whether these effects were the same during both periods before and after HCV cure.

## Materials and methods

### Study setting

ANRS CO22 HEPATHER is an ongoing French nationwide multicenter observational prospective cohort study, which enrolled patients with chronic HCV or HBV infections (ClinicalTrials.gov number NCT01953458). Its main aim is to assess the benefits and risks associated with various treatment strategies for HCV (including DAA) and HBV, and to identify the virological, environmental and social factors predicting the clinical evolution of patients with chronic hepatitis infection.

### Data collection

In the present study, we only focused on patients with chronic HCV infection at enrolment in ANRS CO22 HEPATHER, defined as detectable HCV RNA and positive anti-HCV antibodies. The following patients were excluded from the cohort: HIV co-infected patients, patients already receiving HCV therapy or who had discontinued HCV therapy for less than 3 months at enrolment, minors, persons under legal protection or guardianship, persons prevented from making judicial or administrative decisions, patients with a life expectancy of less than 1 year, and pregnant women.

Patients were enrolled from August 2012 to December 2015 in 32 centers and will be followed until 2024. For the present study, we used data collected in ANRS CO22 HEPATHER until 2018 (*i.e*., during the first 4 years of follow-up). At the enrolment visit, patients underwent a clinical examination with urine and blood sampling. Their HCV physician also collected sociodemographic and behavioral characteristics using a structured questionnaire. All socio-behavioral, clinical and biological information was recorded using a dedicated electronic case-report form. Clinical and biological data are updated during clinical visits, which are scheduled once a year during the follow-up. Other clinical data (*e.g.*, HCV genotype, diabetes) were retrieved from medical records. More detailed information about the cohort can be found elsewhere.^3^

### Study outcome

Data derived from blood samples during follow-up included platelet count, aspartate aminotransferase and alanine aminotransferase (ALT) levels, which enabled the FIB-4 index^14^ to be computed at each visit during follow-up. The study outcome was the presence of severe liver fibrosis – defined as an FIB-4 >3.25 – as a binary time-varying variable.^14^ The choice to use the FIB-4 index was based on its capacity to accurately predict advanced stages of liver fibrosis even after HCV cure,^15^^,^^16^ and its ability to predict complications such as hepatic decompensation and HCC in HCV-infected patients.^17^

### Explanatory variables

The following variables were tested as potential predictors of the study outcome: sex, age, educational level (≥ secondary school certificate, < secondary school certificate), employment, living with a partner, living in poverty, migrant status, HCV genotype, HCV cure, BMI, diabetes, current and previous tobacco smoking, alcohol consumption, cannabis use, psychoactive substance use (other than cannabis), and coffee consumption (number of cups per day). For the latter, we also created a 5-category variable (0, 1, 2, 3, ≥4 cups/day) to test for a dose-response relationship.

Living in poverty was defined as reporting an average monthly household income <1,015 euros per adult equivalent, which corresponds to the poverty line for France in 2015, as defined by the French National Institute for Statistical and Economic Studies (INSEE).^18^ Migrant status was defined as being not born in France and having at least 1 parent of non-French origin^19^. A BMI of <18.5, ≥18.5 and <25, ≥25 and <30, ≥30, was classified as underweight, normal weight, overweight, and obesity, respectively.^20^ Alcohol consumption was classed into 4 categories: abstinent with no history of unhealthy use; abstinent with a history of unhealthy use; moderate use; unhealthy use. Unhealthy alcohol use was defined as >2 and >3 standard drinks per day for women and men, respectively.^21^ HCV cure status was assessed at each follow-up visit and was defined as having a sustained virological response at least 12 weeks after the end of DAA treatment.

All the explanatory variables were measured at enrolment, except for HCV cure, which was used as a time-varying variable in the analysis.

### Study population

The study population comprised chronic HCV-infected patients enrolled in the ANRS CO22 HEPATHER cohort, who had at least one FIB-4 assessment during the 4 years of follow-up and for whom data on the study’s main socio-behavioral explanatory variables (coffee and alcohol consumption, employment, and educational level) were collected at enrolment.

### Ethical considerations

The ongoing ANRS CO22 HEPATHER cohort study is conducted according to the ethical principles set out in the Helsinki Declaration (59^th^ General Assembly of the World Medical Association, Seoul, Korea; October 2008) and French law for biomedical research. It was approved by the ‘CPP Ile de France 3’ Ethics Committee (Paris, France) and the French National Agency for the Safety of Medicines and Health Products (ANSM). Patients could be enrolled only after providing written informed consent, and collected data were centralized using a dedicated information system.

### Statistical analyses

We described the characteristics of the study population at cohort enrolment visit using medians (IQR) or frequencies and percentages, according to the type of variable analyzed (*i.e*., continuous or categorical). The main sociodemographic characteristics at enrolment were compared between patients included in the study population and those not included, using a Chi-square test (for categorical variables) or Student’s *t* test (for continuous variables).

Univariable and multivariable mixed-effects logistic regression models were used to estimate the associations between the potential predictors and the longitudinal measures of severe liver fibrosis, while accounting for the possible correlation between these repeated measures. All visits with an available assessment of the study outcome during follow-up were included in the analyses.

We built 2 multivariable models: the first tested coffee consumption as a categorical variable, to assess whether a dose-response relationship existed (Model 1); the second tested coffee consumption as a continuous variable (Model 2). We chose not to include the variable ‘living in poverty’ in either model, in favor of the other proxies of social conditions - employment and educational level - as they had fewer missing values. Potential predictors with a liberal *p* value <0.20 (Wald test) in the univariable analyses were considered eligible to enter the multivariable models. The final multivariable models were built using a backward stepwise selection procedure with a significance threshold for *p* values of 0.05 (Wald test).

In the second multivariable model, we tested for significant interactions between HCV cure and the following variables: coffee and alcohol consumption, employment, educational level, diabetes, HCV genotype. Interactions between coffee consumption and the other significant predictors were also tested for.

We also estimated the adjusted population attributable fractions (PAFs) for the modifiable clinical and socio-behavioral risk factors, which were included in Model 1 (see above). For each of these factors, we used the punafcc Stata command,^22^ which allows the baseline observed scenario ("Scenario 0") and a fantasy scenario ("Scenario 1") to be compared. In this scenario, the factor in question is assumed to be set to the reference value, while all other explanatory variables in the multivariable model are assumed to remain the same. The PAF represents the fraction of the severe fibrosis burden attributable to being in Scenario 0 instead of Scenario 1.

We also performed stratified analyses according to HCV cure status (*i.e*., cured or not cured), to compare the effects of the predictors included in Model 2 (see above) on severe fibrosis before and after HCV cure. All visits (*i.e.*, pre- and post-cure) of patients cured during follow-up were included in these 2 analyses, respectively. All visits of patients not cured during the 4 years of follow-up were included in the ‘before HCV cure’ analysis.

All statistical analyses were performed using Stata software, version 14.2 for Windows (StataCorp, College Station, Texas, USA).

## Results

Of the 10,698 patients with chronic HCV infection enrolled in the ANRS CO22 HEPATHER cohort, 9,692 were included in the present study population, accounting for 24,687 visits over the first 4 years of follow-up. Of these, 48.5% took place after HCV cure. The median (IQR) number of follow-up visits per patient was 2 (1-3).

No significant differences were found between patients included in the study population and those not included, in terms of the main sociodemographic characteristics at enrolment, except for age (included patients were 2 years older on average, data not shown).

At enrolment, median (IQR) age was 56 (50-64) years, 44.2% of the study population were women, 30.7% reported drinking ≥3 cups of coffee/day, and 57.0% were unemployed (Table 1). With regard to alcohol consumption, 5.9% of men reported unhealthy alcohol use *vs.* 2.6% of women, and 41.2% of men reported moderate alcohol use *vs.* 36.5% of women. More than half (59.0%) had received DAAs during follow-up, with a sustained virological response rate of 96.2%. The distribution of patients according to the 3 categories: FIB-4 >3.25 (severe liver fibrosis stage), 1.45≤FIB-4≤3.25 (indeterminate fibrosis stage), and FIB-4 <1.45 (low fibrosis stage) was, respectively, 26.0%, 40.1% and 33.9% at enrolment and 16.9%, 41.3% and 41.8% at the last available visit. The results of the univariable and multivariable analyses of the associations of potential predictors with severe fibrosis are presented in Table 1.

**Table 1:** **Factors associated with severe liver fibrosis (FIB-4 >3.25) in longitudinal analysis, univariable and multivariable mixed-effects logistic regression models, ANRS CO22 HEPATHER cohort (9,692 patients, 24,687 visits)**.

	Descriptive statistics^1^	Univariable analyses		Multivariable analysis				
				Model 1			Model 2	
	n (%) or median (IQR)	OR (95% CI)	*p* value	aOR (95% CI)	*p* value	PAF % (95% CI)	aOR (95% CI)	*p* value
Coffee consumption (number of cups/day) (continuous)	1 (0-3)	0.50 (0.47-0.53)	<0.001				0.42 (0.38-0.46)	<0.001
Coffee consumption (number of cups/day) (categories)			**<0.001**		**<0.001**			
0 (ref.)	2,781 (28.7)	1		1				
1	2,120 (21.9)	0.74 (0.58-0.94)	0.013	0.47 (0.35-0.63)	<0.001			
2	1,811 (18.7)	0.30 (0.24-0.39)	<0.001	0.18 (0.13-0.25)	<0.001			
3	1,209 (12.5)	0.12 (0.09-0.17)	<0.001	0.07 (0.05-0.11)	<0.001			
4	1,771 (18.3)	0.07 (0.05-0.09)	<0.001	0.04 (0.03-0.06)	<0.001			
HCV cure status								
No (ref.)	3,977 (41.0)	1		1			1	
Yes	5,715 (59.0)	0.12 (0.10-0.14)	<0.001	0.13 (0.11-0.15)	<0.001		0.10 (0.08-0.13)	<0.001
Interaction: Coffee consumption (number of cups/day) (continuous) ∗ HCV cure							1.19 (1.08-1.31)	0.001
Cannabis use								
No (ref.)	6,190 (63.9)	1						
Yes	3,502 (36.1)	0.65 (0.54-0.79)	<0.001					
Tobacco smoking								
No (ref.)	3,474 (35.9)	1						
Yes	6,216 (64.1)	0.80 (0.66-0.96)	0.017					
Current alcohol consumption^2^			**<0.001**		**<0.001**			**<0.001**
Abstinent, no history of unhealthy use (ref.)	4,008 (41.4)	1		1			1	
Abstinent, with history of unhealthy use	1,467 (15.1)	2.15 (1.65-2.80)	<0.001	3.63 (2.61-5.03)	<0.001	15 (13 ; 17)	3.63 (2.62-5.04)	<0.001
Moderate use	3,788 (39.1)	0.48 (0.39-0.58)	<0.001	0.74 (0.58-0.95)	0.019	-11 (-21 ; -1)	0.74 (0.58-0.95)	0.019
Unhealthy use	429 (4.4)	2.14 (1.38-3.31)	0.001	4.63 (2.75-7.80)	<0.001	5 (4 ; 5)	4.66 (2.77-7.85)	<0.001
Current or previous use of psychoactive substance (other than cannabis)								
No (ref.)	6,257 (64.6)	1						
Yes	3,435 (35.4)	0.79 (0.65-0.95)	0.013					
Diabetes								
No (ref.)	8,380 (86.5)	1		1			1	
Yes	1,312 (13.5)	5.68 (4.41-7.32)	<0.001	4.28 (3.16-5.79)	<0.001	17 (15 ; 18)	4.27 (3.15-5.77)	<0.001
BMI			**<0.001**					
Normal weight (ref.)	4,884 (50.7)	1						
Underweight	329 (3.4)	0.91 (0.55-1.53)	0.730					
Overweight	3,117 (32.4)	1.36 (1.12-1.67)	0.003					
Obesity	1,297 (13.5)	1.91 (1.45-2.50)	<0.001					
HCV genotype			**<0.001**		**<0.001**			**<0.001**
1 (ref.)	6,302 (65.0)	1		1			1	
2	644 (6.6)	0.76 (0.53-1.10)	0.151	0.56 (0.36-0.87)	0.010		0.55 (0.35-0.86)	0.009
3	1,280 (13.2)	2.77 (2.12-3.61)	<0.001	7.27 (5.26-10.1)	<0.001		7.25 (5.24-10.03)	<0.001
4	1,248 (12.9)	0.80 (0.61-1.06)	0.120	1.04 (0.75-1.46)	0.795		1.04 (0.74-1.45)	0.822
5/6/7	218 (2.2)	0.97 (0.53-1.76)	0.912	0.54 (0.27-1.10)	0.089		0.53 (0.26-1.08)	0.079
Sex								
Man (ref.)	5,412 (55.8)	1		1			1	
Woman	4,280 (44.2)	0.68 (0.57-0.82)	<0.001	0.33 (0.26-0.42)	<0.001		0.33 (0.26-0.42)	<0.001
Age (continuous)	56 (50-64)	1.07 (1.06-1.08)	<0.001	1.12 (1.10-1.13)	<0.001		1.12 (1.10-1.13)	<0.001
Migrant status								
No (ref.)	7,131 (73.6)	1						
Yes	2,559 (26.4)	1.37 (1.12-1.68)	0.002					
Living with a partner								
No (ref.)	4,202 (43.4)	1						
Yes	5,480 (56.6)	0.84 (0.70-1.01)	0.061					
Unemployed								
No (ref.)	4,168 (43.0)	1		1			1	
Yes	5,524 (57.0)	4.70 (3.90-5.67)	<0.001	1.69 (1.32-2.16)	<0.001	29 (18 ; 39)	1.69 (1.32-2.16)	<0.001
Educational level								
≥secondary school certificate (ref.)	4,412 (45.5)	1		1			1	
<secondary school certificate	5,280 (54.5)	2.18 (1.82-2.61)	<0.001	1.50 (1.20-1.86)	<0.001	21 (11 ; 30)	1.50 (1.21-1.87)	<0.001

Model 1: with coffee consumption as a 5-category variable (without interaction); Model 2: with coffee consumption as a continuous variable and interaction with HCV cure. For variables with more than two categories, global *p* values appear in bold type.

aOR, adjusted odds ratio; BMI, body mass index; CI, confidence interval; FIB-4, fibrosis-4; IQR, interquartile range; OR, odds ratio; PAF, population attributable fraction.

1 All the explanatory variables were measured at enrolment, except for ‘HCV cure status’, which was used as a time-varying variable in the analyses. Descriptive statistics are given at last available visit for HCV cure variable.

2 Unhealthy alcohol use was defined as >2 and >3 standard drinks per day for women and men, respectively.

**Fig. 1:**
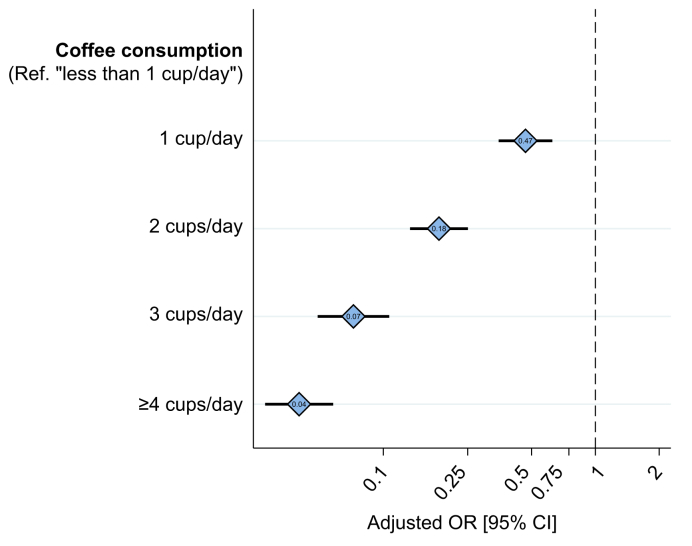
Relationship between coffee consumption and severe liver fibrosis (FIB-4 >3.25), ANRS CO22 HEPATHER cohort (9,692 patients, 24,390 visits). Adjusted for variables presented in Table 1, Model 1. FIB-4, fibrosis-4; OR, odds ratio.

After multivariable adjustment, a significant inverse dose-response relationship was found between the 5-category coffee consumption variable (with no consumption as the reference category) and severe fibrosis (Table 1 and Fig. 1). More specifically, the risk of severe fibrosis significantly diminished as the number of cups consumed per day increased: from a 53% reduced risk (adjusted odds ratio (aOR) 0.47; 95% CI 0.35-0.63) for 1 cup/day to 96% (aOR 0.04; 95% CI 0.03-0.06) for ≥4 cups/day (Table 1, Model 1). When coffee consumption was alternatively used as a continuous variable in the second multivariable model, the risk of severe fibrosis decreased by 58% for each additional cup (aOR 0.42; 95% CI 0.38-0.46) (Table 1, Model 2).

Men and individuals with HCV genotype 3 (*vs.* genotype 1) had a 3- and 7-fold higher risk of severe fibrosis, respectively (Table 1, Model 1). Persons who were unemployed (aOR 1.69; 95% CI 1.32-2.16) and those with a lower educational level (< secondary school certificate) (aOR 1.50; 95% CI 1.20-1.86) were both at higher risk of severe fibrosis. Compared with alcohol abstinent individuals, the risk of severe fibrosis was 3.6/4.6-fold higher in individuals with previous/current unhealthy alcohol use (aOR 3.63; 95% CI 2.61-5.04 and aOR 4.66; 95% CI 2.77-7.85, respectively). HCV-cured patients had an 87% lower risk of severe fibrosis (aOR 0.13; 95% CI 0.11-0.15), while those with diabetes had a 4-fold greater risk (aOR 4.28; 95% CI 3.16-5.79).

Although smoking was identified as a ‘protective’ factor in the univariable analysis, it was no longer significant in the multivariable analysis, perhaps because it was correlated to coffee consumption (*p* <0.001).

None of the interactions tested in the second multivariable model were significant, except that between the number of cups of coffee per day and HCV cure (Table 1, Model 2). The fact that no other interaction was statistically significant suggests that the effects of the other predictors on severe fibrosis were similar before and after HCV cure.

The estimated adjusted PAFs highlighted that the risk factors accounting for the greatest severe fibrosis burden were unemployment (PAF 29%; 95% CI 18%-39%), low education level (PAF 21%; 95% CI 11%-30%), diabetes (PAF 17%; 95% CI 15%-18%), history of unhealthy alcohol use (PAF 15%; 95% CI 13%-17%), and current unhealthy alcohol use (PAF 5%; 95% CI 4%-5%) (Table 1, Model 1).

**Fig. 2:**
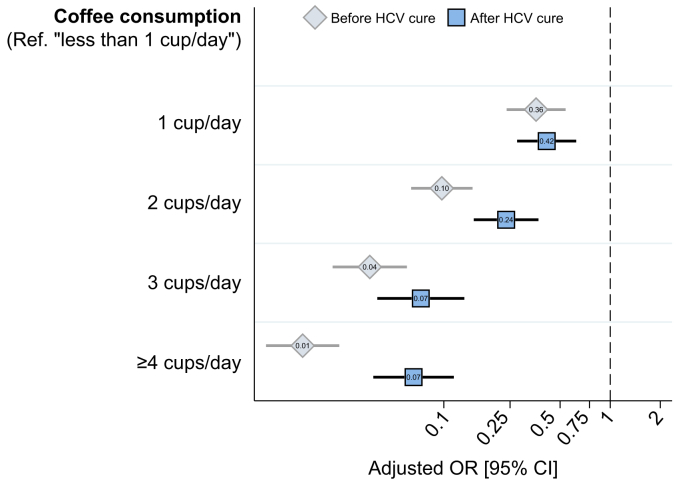
Relationship between coffee consumption and severe liver fibrosis (FIB-4 >3.25) stratified by HCV cure status, ANRS CO22 HEPATHER cohort (9,692 patients, 24,390 visits). Adjusted for variables presented in Table 1, Model 1. FIB-4, fibrosis-4; OR, odds ratio.

The stratified analysis (data not shown) highlighted that the protective effect of coffee consumption on severe fibrosis was significantly greater before HCV cure than after it (aOR 0.34; 95% CI 0.30-0.38 *vs.* aOR 0.48; 95% CI 0.43-0.55 per 1 cup/day increase). This can also be seen in the dose-response relationship (from 0 to ≥4 cups/day) in Fig. 2, which was more significant before HCV cure, mainly due to the lack of additional benefits on regression of severe fibrosis from drinking ≥4 cups/day after HCV cure.

## Discussion

Using longitudinal data from a very large French nationwide prospective cohort implemented at the beginning of the DAA era, the present study explored the associations between severe liver fibrosis (as measured by an FIB-4 >3.25) and lifestyle-related factors, unhealthy behaviors, social conditions, and HCV cure in chronic HCV-infected patients. There are 3 main results: first, a very strong inverse dose-response relationship between coffee consumption and severe fibrosis was observed, with the risk of severe fibrosis halving for each additional cup of coffee consumed per day. In addition, the antifibrotic activity of coffee consumption continued even after HCV cure with major effects observed for ≥3 cups/day. Second, low socioeconomic status, diabetes, and previous and current unhealthy alcohol use were the 3 predictors which accounted for the greatest severe fibrosis burden, and had similar effects before and after HCV cure. These associations probably reflect fewer opportunities for timely HCV screening and referral to HCV care for these vulnerable groups. Third, HCV cure was the main clinical factor; it was associated with an 87% lower risk of severe fibrosis.

In patients with liver disease, there is compelling evidence for the hepatoprotective effects of coffee consumption, specifically better liver function (ALT activity in particular), and a reduced risk of fibrosis and cirrhosis,^23^ liver cancer,^11^ and mortality.^24^ Estimates from a meta-analysis indicate that the risk of liver cancer is 40% lower in individuals with elevated coffee consumption.^11^ Most studies to date have found that the consumption threshold for significant benefits on liver health and mortality is ≥3 cups per day. The dose-response relationship in our study is consistent with those found in a previous study showing an inverse association between coffee consumption and both elevated levels of serum ALT and gamma glutamyltransferase in the general population.^25^ This relationship was also suggested by various meta-analyses which found that increasing coffee consumption may substantially reduce the risk of cirrhosis^26^ and of liver cancer, the latter by 15% per 1 cup/day increase.^27^

The dose-response relationship observed persisted after HCV cure, with a daily consumption of ≥3 cups bringing the greatest protective effects against severe liver fibrosis (Fig. 2). We wonder whether this relationship existed because the FIB-4 index is more predictive of liver cancer than other markers of fibrosis,^28^ especially given that the risk of liver cancer also has a dose-response relationship to coffee consumption.

The benefits observed with increased levels of coffee consumption may be attributable to caffeine and other polyphenols, which display multiple hepatoprotective effects. The main mechanism involved in the protective effect of coffee extracts on liver fibrosis and the onset of cirrhosis, is the inhibition of hepatic stellate cell activation. Indeed, coffee, or more particularly its specific compounds (such as caffeine, chlorogenic acids, phenolic compounds and diterpenes) appear to reduce not only hepatic stellate activation, which is involved in fibrogenesis and hepatic inflammation, but also fatty acid synthesis, which is implicated in steatogenesis.^29^ Moreover, coffee has been found to induce apoptosis and increased hepatic antioxidant capacity, both of which are involved in carcinogenesis.^30^ Another mechanism through which coffee presents its antifibrotic activity, is its potential to predict a higher microbial diversity, which is associated with better liver function.^31^

Coffee drinking has also been associated with a lower risk of all-cause death,^32^ including in patients with HIV and hepatitis C.^12^^,^^33^ Moreover, it seems to reduce the negative effects of unhealthy alcohol use on liver fibrosis in HCV-infected individuals.^34^

Consistent with previous research, we found that unhealthy alcohol use^9^ and social condition proxies^35^ – expressed in our study by unemployment and a low educational level – were also significant independent predictors of severe fibrosis. This suggests that negative social conditions, as well as current or past unhealthy alcohol use, constitute missed opportunities for prompt HCV screening and referral for HCV care, resulting in more advanced liver disease by the time treatment is initiated. Negative social conditions may also capture unmeasured effects of specific lifestyles associated with social vulnerability, such as unhealthy nutrition, alcohol consumption, underweight, and overweight.^36^ The lack of any interaction between HCV cure and these variables in our study suggests that such missed opportunities may continue to negatively impact liver fibrosis progression even after HCV cure.

Furthermore, we found that moderate alcohol consumption was associated with a lower risk of severe fibrosis. A possible explanation for this is that low/moderate alcohol consumption can be regarded as a proxy of healthier lifestyle-related behaviors (*e.g.* high-quality food, exercise) and high social status, which were not completely captured by the variables included in our models.^37^ In our study population, moderate users of alcohol were less likely to have type 2 diabetes (10.5%) than abstinent participants with or without a history of unhealthy alcohol use (16.0%). Furthermore, the percentage of tobacco smokers was lower than that of persons with unhealthy alcohol use (42.8% *vs.* 73.9%) and lower than the percentage of abstinent participants with a history of unhealthy alcohol use (58.0%).

The association we found between HCV cure following DAAs and an 87-90% (Model 1-Model 2, Table 1) lower risk of severe fibrosis was expected, and is consistent with previous research showing that HCV cure significantly reduces liver stiffness after virological response to DAAs.^38^ Moreover, HCV cure following DAAs was associated with a lower risk of HCC and mortality, which reflects findings in a previous study of the same cohort.^3^

The association between diabetes and severe fibrosis in our study reflects previous results showing that diabetes can accelerate fibrosis progression.^39^ Furthermore, the lack of interaction between diabetes and HCV cure confirms that its effect on severe fibrosis persists after sustained virological response. Just as for patients with past or current unhealthy alcohol use, this underlines the importance of scheduling regular clinical follow-up and liver function assessment for diabetic patients even after HCV cure.

The association between being a woman and a lower risk of severe fibrosis, has not been clearly identified in other studies, although it has previously been shown that women may be protected from liver fibrosis progression during the pre-menopausal period.^40^ Although the average age of women in our study corresponded to the post-menopausal period, we hypothesize that the benefits of the pre-menopausal period on liver fibrosis persist for a relatively long period, leading to less progression of severe fibrosis in women than in men.

HCV genotype 3 was associated with severe fibrosis in our study, which is consistent with recent research showing that individuals with this genotype have almost a 7-fold greater risk of severe fibrosis.^41^ This genotype is also more frequent in people who inject drugs, a population that generally experiences delayed engagement in HCV care, and that has higher prevalences of unhealthy alcohol use and severe fibrosis before treatment initiation.^42^

This study has several strengths, including the large size of the study population and the longitudinal collection of data in the real-world setting of an observational hospital-based cohort. Moreover, it is the first study to explore modifiable socio-behavioral risk factors (such as socioeconomic indicators and lifestyle-related unhealthy behaviors) as potential correlates of severe liver fibrosis, both before and after HCV cure. The study’s main limitation is that behaviors were self-reported. Accordingly, behaviors perceived as less socially acceptable (*e.g*., alcohol consumption) may have been under-reported, causing a proportion of those with unhealthy behaviors to be unidirectionally misclassified as having healthy behaviors. However, the effect of any such underreporting would only lead to an underestimation of the strength of the association between each behavior and the outcome. Moreover, thanks to the large size of the study population, significant effects were revealed even if they may have been underestimated.

To conclude, promoting coffee consumption and monitoring liver fibrosis progression even after HCV cure are crucial strategies for patients with low socioeconomic status, current or past unhealthy alcohol use and diabetes. It is not known whether fibrosis regression after HCV cure results in a lower risk of HCC over the long term. As the risk of HCC can change over time because of non-HCV-related risk factors, such as older age, unhealthy alcohol use, obesity and diabetes, we believe that liver fibrosis monitoring – using the FIB-4 index, which is a simple and easy-to-use fibrosis marker – should be offered to all patients with these factors, irrespective of their fibrosis stage at the end of treatment. Innovative HCV care models for the most socially vulnerable individuals and interventions for healthier lifestyles are needed to reinforce HCV cure effects on liver health.

## Financial support

The ANRS CO22 HEPATHER cohort received financial support from INSERM-ANRS MIE (France Recherche Nord & Sud Sida-VIH Hépatites, Maladies Infectieuses Emergentes), the French ANR (Agence Nationale de la Recherche), the French DGS (Direction Générale de la Santé), Merck Sharp and Dohme, 10.13039/100015756Janssen-Cilag, Gilead, 10.13039/100006483Abbvie, 10.13039/100002491Bristol-Myers Squibb and 10.13039/100004337Roche.

## Authors’ contributions

Study concept and design: PC, FC, EDA, SP, HF, CP; Data acquisition: FC, MB, VDL, GPP, CD, SP, HF; Analysis and interpretation of data: PC, VDB, TB, FM, CP; Drafting of the manuscript: PC, CP; Critical revision of the manuscript for important intellectual content: FC, VDB, MB, TB, VDL, GPP, MB, CC, CD, EDA, FM, SP, HF; Statistical analysis: VDB, CP; Obtaining funding: PC, FC, MB, CC, HF; Administrative, technical, or material support: VDB, MB, CC, CD; Study supervision: PC, FC, SP, HF, CP. All the authors approved the final version of the manuscript, and agree to be accountable for all aspects of the work in ensuring that questions related to the accuracy or integrity of any part of the article are appropriately investigated and resolved.

## Data availability statement

Data are available upon request to the scientific committee of the ANRS CO22 HEPATHER cohort, which includes the authors of the manuscript (contact: fabrice.carrat@iplesp.upmc.fr).

## Conflict of interest

Stanislas Pol has served as a speaker, a consultant and an advisory board member for Janssen, Gilead, Roche, MSD, Abbvie, Biotest, Shinogi, Vivv, and LFB. He has received research funding from Gilead, Abbvie, Roche and MSD not connected to the present work. Fabrice Carrat reports grants from INSERM-ANRS during the implementation of this study and personal fees from Imaxio, outside the submitted work. Patrizia Carrieri received research grants from MSD and Intercept unrelated to this work. Georges-Philippe Pageaux received lecturing fees from Gilead, Abbvie, outside the submitted work. Victor De Lédinghen has received consulting and/or lecturing fees from Gilead, AbbVie, Echosens, Intercept Pharma, Super-Sonic Imagine, Indivior, Spimaco, Pfizer, Bristol-Myers Squibb, Myr-Pharma. Marc Bourlière reports grants and personal fees from AbbVie, grants and personal fees from Gilead, personal fees from MSD, personal fees from Janssen, personal fees from Boehringher Ingelheim, personal fees from intercept, personal fees from BMS, outside the submitted work. Hélène Fontaine reports personal fees and invitations for medical meeting from Gilead, Abbvie, BMS, MSD, Janssen, MSD outside this work. The other authors have no conflict of interest to declare.

Please refer to the accompanying ICMJE disclosure forms for further details.

## ANRS/AFEF HEPATHER Study group

### Investigators

Laurent Alric, Delphine Bonnet, Virginie Payssan-Sicart, Chloe Pomes (CHU Purpan, Toulouse, France), Fabien Zoulim, Marianne Maynard, Roxane Bai, Lucie Hucault, François Bailly (Hospices Civils de Lyon, Lyon, France), François Raffi, Eric Billaud, David Boutoille, Maeva Lefebvre, Elisabeth André-Garnier (Hôpital Hôtel-Dieu, Nantes, France), Paul Cales, Isabelle Hubert, Adrien Lannes, Françoise Lunel, Jérôme Boursier (CHU Angers, Angers, France), Tarik Asselah, Nathalie Boyer, Nathalie Giuily, Corinne Castelnau, Giovanna Scoazec (Hôpital Beaujon, Clichy, France), Stanislas Pol, Hélène Fontaine, Emilie Rousseaud, Anaïs Vallet-Pichard, Philippe Sogni (Hôpital Cochin, Paris, France), Victor de Lédinghen, Juliette Foucher, Jean-Baptiste Hiriart, Jancell M’Bouyou, Marie Irlès-Depé (Hôpital Haut-Lévêque, Pessac, Bordeaux, France), Marc Bourlière, Si Nafa Si Ahmed, Valérie Oules (Hôpital Saint Joseph, Marseille, France), Albert Tran, Rodolphe Anty, Eve Gelsi, Régine Truchi (CHU de Nice, Nice, France), Dominique Thabut, Saloua Hammeche, Joseph Moussali (Hôpital de la Pitié Salpétrière, Paris, France), Xavier Causse, Barbara De Dieuleveult, Brahim Ouarani, Damien Labarrière (CHR La Source, Orléans, France), Nathalie Ganne, Véronique Grando-Lemaire, Pierre Nahon, Séverine Brulé, Betul ULKER (Hôpital Jean Verdier, Bondy, France), Dominique Guyader, Caroline Jezequel, Audrey Brener, Anne Laligant, Aline Rabot, Isabelle Renard (CHU Rennes, Rennes, France), François Habersetzer, Thomas F. Baumert, Michel Doffoel, Catherine Mutter, Pauline Simo-Noumbissie, Esma Razi (Hôpitaux Universitaires de Strasbourg, Strasbourg, France), Jean-Pierre Bronowicki, Hélène Barraud, Mouni Bensenane, Abdelbasset Nani, Sarah Hassani-Nani, Marie-Albertine Bernard (CHU de Nancy, Nancy, France), Georges-Philippe Pageaux, Dominique Larrey, Magda Meszaros (Hôpital Saint-Eloi, Montpellier, France), Sophie Metivier, Christophe Bureau, Thibault Morales, Jean Marie Peron, Marie Angèle Robic (CHU Purpan, Toulouse, France), Thomas Decaens, Bruno Froissart, Marie-Noelle Hilleret, Charlotte Costentin, Théophile Gerster (CHU de Grenoble, Grenoble, France), Ghassan Riachi, Odile Goria, Fatima Paris, Hélène Montialoux (CHU Charles Nicolle, Rouen, France), Vincent Leroy, Giuliana Amaddeo, Anne Varaut, Mélanie Simoes, Rachida Amzal (Hôpital Henri Mondor, Créteil, France), Olivier Chazouillières, Tony Andreani, Bénédicte Angoulevant, Azeline Chevance, Lawrence Serfaty (Hôpital Saint-Antoine, Paris, France), Jean-Charles Duclos Vallée, Didier Samuel, Teresa Antonini, Audrey Coilly, Mariagrazia Tateo (Hôpital Paul Brousse, Villejuif, France), Armand Abergel, Maud Reymond, Chanteranne Brigitte, Buchard Benjamin, Léon Muti (Hôpital Estaing, Clermont-Ferrand, France), Claire Geist, Guillaume Conroy, Raphaëlle Riffault (Centre Hospitalier Régional, Metz, France), Isabelle Rosa, Camille Barrault, Laurent Costes, Hervé Hagège (Centre Hospitalier Intercommunal, Créteil, France), Véronique Loustaud-Ratti, Paul Carrier, Maryline Debette-Gratien, (CHU Limoges, Limoges, France), Philippe Mathurin, Guillaume Lassailly, Elise Lemaitre, Valérie Canva, Sébastien Dharancy, Alexandre Louvet (CHRU Claude Huriez, Lille, France), Anne Minello, Marianne Latournerie, Marc Bardou, Thomas Mouillot (Dijon University Hospital, Dijon, France), Louis D’Alteroche, Didier Barbereau, Charlotte Nicolas, Laure Elkrief, Anaïs Jaillais (CHU Trousseau, 37044 Tours, France), Jérôme Gournay, Caroline Chevalier, Isabelle Archambeaud, Sarah Habes (CHU de Nantes, Nantes, France), Isabelle Portal (CHU Timone, Marseille, France), Moana Gelu-Simeon, Eric Saillard, Marie-Josée Lafrance, Lucie Catherine (CHU de Pointe-à-Pitre, Pointe-à-Pitre, Guadeloupe).

### Methodology and Coordinating Centre

Fabrice Carrat (coordinator), Frederic Chau, Céline Dorival, Isabelle Goderel, Clovis Lusivika-Nzinga, Marc-Antoine Bellance, Jonathan Bellet, Priscilla Monfalet, Jessica Chane-Teng, Sephora Bijaoui, Grégory Pannetier, François Téoulé, Jérôme Nicol, Rafika Bekhti (Sorbonne University & INSERM U1136 - IPLESP, Paris, France).

### Scientific Committee

**Voting members:** Marc Bourlière (Hôpital St Joseph, Marseille), Jérôme Boursier (CHU Angers, Angers, France), Fabrice Carrat (Scientific Coordinator, Hôpital Saint-Antoine, Paris, France), Patrizia Carrieri (INSERM U912, Marseille, France), Elisabeth Delarocque-Astagneau (Inserm UMR1181, Paris), Victor De Lédinghen (Hôpital Haut-Lévêque, Pessac, Bordeaux, France), Céline Dorival (UPMC & INSERM U1136, Paris, France), Hélène Fontaine (Hôpital Cochin, Paris, France), Slim Fourati (Hôpital Henri Mondor, Créteil, France), Chantal Housset (Inserm UMR-S938 1 IFR65, Paris), Dominique Larrey (Hôpital Saint-Eloi, Montpellier, France), Pierre Nahon (Hôpital Jean Verdier, Bondy, France), Georges-Philippe Pageaux (Hôpital Saint-Eloi, Montpellier, France), Ventzislava Petrov-Sanchez (ANRS, Paris, France), Stanislas Pol (Principal Investigator, Hôpital Cochin, Paris, France), Mathias Bruyand (Agence Nationale de Santé Publique, Saint Maurice, France), Linda Wittkop (ISPED-INSERM U897, Bordeaux, France), Fabien Zoulim (Hospices Civils de Lyon, Lyon, France), Jessica Zucman-Rossi (Inserm U674/1162, Paris). **Non-voting members:** Marianne L’hennaff (ARCAT-TRT-5-CHV, France), Michèle Sizorn (SOS hépatites, France); one representative of INSERM-ANRS-MIE Pharmacovigilance team, Paris, France (Anaïs Boston, Alpha Diallo), Carole Cagnot (INSERM-ANRS-MIE, Paris, France), one member of Inserm Transfert, Paris, France (Alice Bousselet, Mireille Caralp), and one representative of each pharmaceutical company (MSD, Gilead, Abbvie).
